# Proteins from Edible Mushrooms: Nutritional Role and Contribution to Well-Being

**DOI:** 10.3390/foods14183201

**Published:** 2025-09-14

**Authors:** Mariana Ionescu, Mirela-Nicoleta Dincă, Mariana Ferdeș, Bianca-Ștefania Zăbavă, Gigel Paraschiv, Georgiana Moiceanu

**Affiliations:** 1 Department of Biotechnical Systems, Faculty of Biotechnical Systems Engineering, National University of Science and Technology Politehnica Bucharest, 060042 Bucharest, Romania; mariana.ionescu@upb.ro (M.I.); mferdes@upb.ro (M.F.); gigel.paraschiv@upb.ro (G.P.); 2 Department of Entrepreneurship and Management, Faculty of Entrepreneurship, Business Engineering and Management, National University of Science and Technology Politehnica Bucharest, 060042 Bucharest, Romania; georgiana.moiceanu@upb.ro

**Keywords:** edible mushrooms, proteins, food industry, medicine

## Abstract

Edible mushrooms represent great promise for the future of food and medicine due to their excellent nutritional, functional, and therapeutic properties. Macrofungi synthesize numerous bioactive compounds, among which proteins stand out for their remarkable diversity, both in terms of structure and their nutritional and functional roles. Fungi from the phylum Basidiomycota have a high protein content, characterized by a complete and balanced amino acid composition. Proteins and peptides from mushrooms have both nutritional and functional roles, with numerous health benefits, such as antimicrobial, antiviral, antioxidant, anticancer, hypotensive, angiotensin-converting enzyme (ACE) inhibition, immunomodulatory, and enzymatic activities. Functional proteins include lectins, immunomodulatory proteins, enzymes (laccase, cellulase, ribonuclease), enzyme inhibitors, ribosome-inactivating proteins, and hydrophobins. In addition to traditional cultivation, mushrooms can be grown as mycelium on solid substrates or in submerged culture, followed by protein separation and extraction. The main trends in protein biosynthesis from Basidiomycota involve both improving the properties of the producing strains and optimizing the cultivation methods in submerged culture and on solid substrates. Moreover, new techniques in the fields of genomics, proteomics, and metabolomics will enable increasingly promising results. This paper provides a systematic overview of the types and properties of proteins from edible mushrooms, with a focus on the main beneficial effects of their consumption.

## 1. Introduction

In the context of constant population growth (in 2050, it will exceed 9 billion), especially in certain areas of the globe, the need for nutrients will also increase, particularly high-quality proteins, along with sugars, lipids, vitamins, enzymes, minerals, and various bioactive compounds that can be obtained from plant, animal, algae, or microorganism sources. In this context, it is absolutely necessary to find new sources of high-quality protein that can be produced in limited spaces and in a short time, at the lowest possible cost.

Macrofungi belonging to the phylum Basidiomycota, commonly called mushrooms, have been known since ancient times for their nutritional, medicinal, and dietary properties, having been consumed since antiquity in countries such as China, the Roman Empire, Greece, India, and others. In the present day, the largest quantities of edible and medicinal are obtained in China, Japan, the USA, Canada, and India [1,2].

There are approximately 2000 known species of edible mushrooms, but the most cultivated and consumed are *Agaricus bisporus*, *Pleurotus ostreatus*, *Pleurotus eryngii*, *Lentinula edodes*, *Volvariella volvacea*, *Flammulina velutipes*, *Hypsizygus stessulatus*, and others [3]. Of these, 700 species are recognized as medicinal, edible, and safe species [4] and contain a wide range of compounds that are valuable from both a nutritional and functional perspective. Recently, numerous studies have been conducted on the characterization of compounds with nutritional and medicinal value and the understanding of their mechanism of action for the purpose of their use [5]. Numerous recently discovered bioactive substances have been shown to have a significant influence on the prevention and improvement of certain diseases, being used in naturally produced pharmaceuticals.

In international cuisine, mushrooms are considered a delight with excellent nutritional properties, a naturopathic food with therapeutic characteristics; in addition, they are accessible to large groups of people both in terms of their geographical distribution and price [6]. Their attractive aroma; high protein, fiber, mineral, and vitamin contents; low fat and cholesterol content; and low calorie content are some of their appealing characteristics [7].

Edible mushrooms have a high protein content (between 19% and 40%), similar to that in animal proteins (pork or beef) and soybeans and higher than the protein concentration in plants [8].

In addition, mushroom cultivation is relatively straightforward, the development time is shorter than that for plants and animals, and it is a possibility to use a wide variety of substrates. Various by-products and waste from the agri-food industry and other sectors can replace the traditional substrate, making edible mushroom cultivation part of the circular economy. Mushrooms can also be cultivated to obtain mycelium, in submerged cultures (SCs) or on solid substrates—solid-state cultivation (SSC)—reducing growth times and the influence of various disruptive factors.

This article provides a systematic overview of the types and properties of proteins in edible mushrooms, aiming to summarize the main beneficial effects of their consumption. Although there are numerous syntheses on bioactive compounds from mushrooms [9,10,11,12], very few discuss the diversity of the proteins in mushrooms, their types, and their beneficial activities for health [5,13]. In most articles, the focus is on the importance and use of edible (medicinal) mushrooms, where the ensemble of synthesized compounds that can be exploited in the food industry is considered. This synthesis aimed to provide an overview of the latest studies in the field of mushroom proteins with a role in nutrition and medicine. This review has the following key sections: highlighting the main bioactive compounds in edible mushrooms and their role in the body; presenting the types of proteins with different roles in mushrooms: proteins, peptides, and amino acids with nutritional roles, lectins, enzymes, enzyme inhibitors, fungal immunomodulatory proteins, ribosome-inactivating proteins, and hydrophobins; the cultivation of mushrooms as mycelium; and directions for future research.

## 2. The Main Bioactive Compounds in Mushrooms

**Figure 1:**
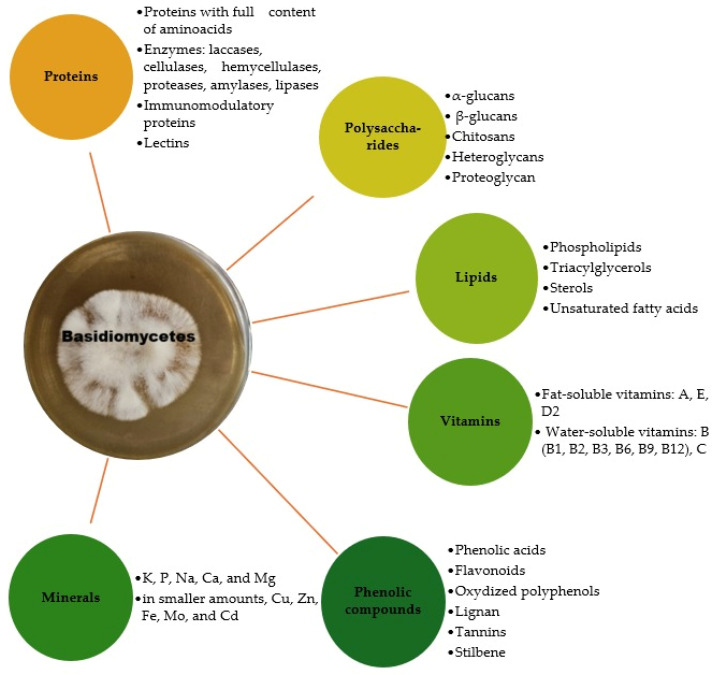
Main bioactive compounds in mushrooms (own creation).

The nutrients and therapeutic substances synthesized by fungi in the Basidiomycota phylum are characterized by great diversity and complexity, but the main groups of compounds with health and nutritional roles can be classified into the following categories: polysaccharides, proteins (including enzymes), lipids, phenolic compounds, terpenes and terpenoids, nucleotides, vitamins, and minerals [14,15] (Figure 1).

### 2.1. Polysaccharides

The most common fungal polysaccharides are α- and β-glucans, chitosan, heteroglycans, and proteoglycans [3] and can be synthesized intra- or extracellularly, with the former having an energetic or structural role and the latter having a protective or adhesive role for different substrates.

In present, the antioxidant, antimicrobial, antitumor, hypocholesterolemic, and hypoglycemic action of exopolysaccharides (EPSs) is well known, and they are the most intensively studied in the medical field [16]. Some examples of polysaccharides are pleuran (produced by species of the genus *Pleurotus*), lentinan (produced by *Lentinula edodes*), and schizophyllan (*Schizophyllum commune*), with anticancer and immunomodulatory properties [9].

Numerous studies have also demonstrated that EPSs act as dietary fibers and prebiotics, promoting the proliferation of probiotic bacteria in the intestines [17].

Research has also shown that many EPSs have immunostimulatory properties, increasing the immune response. For example, β-glucans, polysaccharide–peptides, and polysaccharide–protein complexes act by increasing the number of lymphocyte cells [18], as well as pro-inflammatory cytokines in mice [19].

Laboratory studies have shown that polysaccharides have anti-hyperlipidemic activity, observed in mice that with induced elevated cholesterol levels [10,20]. Mushrooms also have a blood glucose-lowering effect, as demonstrated in the research by Agunloye et al. and Devi et al., which could be exploited in the treatment of people with diabetes [3,21].

### 2.2. Lipids

Edible mushrooms have a low fat content, ranging between 1.18 and 8.39% on a dry weight basis. Triacylglycerols; saturated and unsaturated fatty acids such as linoleic acid, with antitumor properties; phospholipids; and sterols are predominant. Ergosterol, a precursor of vitamin D, is known for its beneficial effects on the cardiovascular system and lipid metabolism [17,22].

### 2.3. Phenolic Compounds

The phenolic compounds synthesized by edible mushrooms belong to various classes, such as flavonoids, oxidized polyphenols, phenolic acids, stilbenes, lignans, hydroxybenzoic acids, hydroxycinnamic acids, and tannins. These compounds are notable for their antioxidant, anti-inflammatory, antitumor, and hypoglycemic activity, with the potential to prevent degenerative nerve cell diseases and delay the aging process. In the food industry, phenolic compounds contribute to the highly appreciated flavor of mushrooms and are used to inhibit lipid oxidation [3].

### 2.4. Terpenes and Terpenoids

Edible mushrooms synthesize secondary metabolites from the class of terpenes and terpenoids with antioxidant, antitumor, anti-inflammatory, antiviral, and insecticidal properties. These compounds have been identified through gas chromatography–mass spectrometry and thin-layer chromatography analyses and are synthesized by most mushrooms [3].

### 2.5. Vitamins and Minerals

Medicinal mushrooms mainly synthesize fat-soluble vitamins, such as A, E, D2 (ergocalciferol) and provitamin D2 (ergosterol). Also, mushrooms contain significant amounts of water-soluble vitamins such as B vitamins (B1, B2, B3, B6, B9, B12) and vitamin C. Among the minerals present in these mushrooms, the most common are K, P, Na, Ca, and Mg and, in smaller amounts, Cu, Zn, Fe, Mo, and Cd, which makes them an important source of micronutrients with nutritional value [23].

## 3. Mushroom Proteins and Their Properties

In the human body, proteins are basic structural units that ensure the normal development and functioning of the cells and also have multiple functions in metabolism: a catalytic role, enzyme inhibition, hormonal function, participation in the activity of the immune system and the transport of certain substances, and others.

Due to their properties, proteins play an essential role in maintaining cellular physiological activities and homeostasis in the human body, and protein malnutrition causes major metabolic dysfunctions, especially in children [24].

Proteins synthesized by fungi in the Basidiomycota phylum are characterized by their great diversity and uniqueness compared to proteins of animal or plant origin. Their chemical structure differs depending on the number, sequence, and type of amino acids, as well as the molecular conformation (the type and location of physical bonds that are established between the amino acids in the molecule, determining the primary, secondary, or tertiary structure) of these compounds. Their physical, chemical, and biological properties are determined by their structure [25,26].

The protein content of different species of edible mushrooms varies considerably depending on the cultivation conditions, physiological stage of maturity, and type of strain. Thus, for *Agaricus bisporus*, the crude protein content (g/100 g dry matter) varies between 29.64 and 39.8 [27,28]. In the case of *Agaricus blazei* Murrill, a protein content of 33.96% was determined [29]. Li et al. [30] demonstrated that in the case of the mushroom *Lentinula edodes* (Berk.) Sing., the six cultivars studied had 14.87–27.13% protein, and significant differences were observed among different cultivars.

In 2019, Ahlborn et al. measured the mycelial dry biomass and protein content in apple pomace cultures of several mushroom species (*Agrocybe aegerita*, *Lentinula edodes*, *Wolfiporia cocos*) and demonstrated that the highest protein values (%DM) were obtained for *Pleurotus sapidus* strain 8266 and *Pleurotus sajor-caju*, namely 25.4 ± 0.3% DM and 20.9 ± 0.3% DM [31], in which glutamic acid (18% of total amino acids; histidine = 10% of total amino acids) predominated. In 2007, Guo et al. obtained a value of 20.4% protein for fruiting bodies of *P. sapidus* [32].

In addition, the proteins synthesized by edible mushrooms differ greatly in terms of their biological activity. Thus, some proteins have antimicrobial and antiviral action (extract from *Ganoderma pfeifferi* [33]) and insecticidal action (lectins from *Coprinopsis cinerea*, *Xerocomus chrysenteron*, *Clitocybe nebularis*, *Aleuria aurantia*, *Sordaria macrospora*, *Sclerotinia sclerotiorum* [34], and *Clitocybe nebularis* [35]). In study [35] it was demonstrated that *Clitocybe nebularis* synthesizes a protein with an insecticidal role against *Drosophila melanogaster*. Numerous fungi contain proteins from the lectin group with antitumor properties [36], such as *Ganoderma lucidum*, *Grifola frondosa*, *Flammulina velutipes*, *Hericium erinaceum*, *Inocybe umbrinella*, and *Pleurotus ostreatus*. The enzymes in the hydrolase and oxidoreductase classes synthesized by wood-degrading and litter-decomposing species are well known and studied [37].

### 3.1. Proteins with Nutritional Value

Most studies have shown that compared to other plant proteins, the nutritional value of mushroom proteins is one of the highest [38]. Although the protein content and amino acid proportions vary considerably depending on species, stage of development, substrate, and other cultivation conditions, most edible mushrooms are an excellent source of high-quality protein [39].

Traditional sources of protein are represented by animal and vegetable proteins. Each of these types of proteins is characterized by the presence of certain types of amino acids in a certain proportion and by a specific sequence of amino acids in the molecule. The presence and percentage of essential amino acids (EAAs) must also be taken into account. Compared to animal proteins, it is known that plant-derived proteins, although cheaper, do not contain sufficient amounts of EAAs and are therefore considered incomplete, lower-quality proteins that should be supplemented with the deficient amino acids from other sources. Other sources of high-quality protein include algae, microorganisms, and insects, which have lower acceptability and are limited in terms of their accessibility [40].

The protein content (g/100 g dry matter) of mushrooms in the Basidiomycota phylum can vary between 29.64 and 39.8% in *Agaricus bisporus* [27,28,41], is 23% for *Lentinula edodes* [42,43,44], is 25.85–39.3% for *Morchella esculenta* [45,46], and is 29.15–33.1 for *Boletus edulis* [47,48,49]. Compared to these values, Saidi et al. showed that beef has 29.5%, pork 28.2%, and chicken 27.4% [50]. The presence of essential amino acids in mushroom proteins has been demonstrated, and the proportion of EAAs is similar to that found in meat proteins [51]. Among the most appreciated species in terms of their amino acid composition are *Agaricus bisporus*, *Flammulina velutipes*, *Tricholoma matsutake*, and *Pleurotus eryngii* [52].

Mushroom proteins are usually rich in sulfur-containing amino acids (methionine and cysteine), in contrast to proteins from vegetables and cereals. They also have a high content of lysine (a basic amino acid), aspartic acid, and glutamic acid, which gives mushrooms their characteristic umami taste.

**Figure 2:**
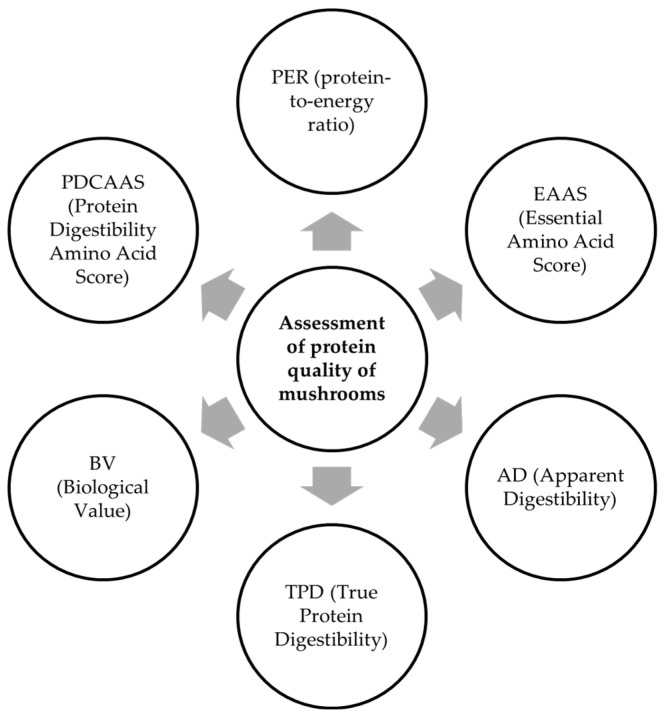
Assessment of protein quality of mushrooms (own creation).

The properties, quality, and digestibility of proteins in mushrooms can be analyzed and compared by considering several parameters that characterize the protein as a whole or each amino acid individually (Figure 2): PER (protein-to-energy ratio), EAAS (Essential Amino Acid Score), AD (Apparent Digestibility), TPD (True Protein Digestibility), BV (Biological Value), and PDCAAS (Protein-Digestibility-Corrected Amino Acid Score) [8]. These properties provide a comprehensive overview of the nutritional values of a particular species or of mushrooms as a whole.

1. The protein-to-energy ratio provides information about the energy value of mushroom protein, which is comparable to that of animal protein and considerably higher than that of vegetable protein (oats and rice) [8]. Thus, according to González et al., species belonging to the *Agaricus* genus have PER values in the range of 0.7–0.9 g kcal^−1^, similar to beef jerky [8]. Different species of *Pleurotus* had PER values of 0.59–0.98 g kcal^−1^ compared to 0.034 (oats) and 0.018 (white rice) [53,54]. Therefore, the consumption of mushrooms with a low calorie content and a high protein percentage is recommended for people who want to lose weight healthily.
2. The Essential Amino Acid Score measures the proportion of each essential amino acid in the protein compared to a standard complete protein. According to Bach et al., almost all essential amino acids in the selected species of *Agaricus*, *Pleurotus*, *Flamulina*, and *Lentinus* had a score higher than 1 mg in 1 g of protein, meaning that the amino acid requirements are met according to the recommended essential amino acid profile for adults [53].
3. Another characteristic of protein is Protein Digestibility, which measures the amount of protein available for absorption after the digestion process and is estimated from dietary, fecal or ileal, and urinary nitrogen values. The amount of ingested protein that is available for absorption represents the Apparent Digestibility and is calculated as the difference between dietary N and fecal N, relative to dietary N. For a more accurate calculation, TPD (True Protein Digestibility) is determined, in which AD is corrected with the mandatory value of fecal N, which is subtracted from fecal N. Some studies have reported the lowest TPD values of approximately 43% for *Pleurotus sajor-caju* or over 80% for *Agaricus macrosporus* [55]. Other studies have shown that the TPD of mushrooms ranges from 72% to 83%, similar to that for soybean (74%) and rice (82%) but lower than that for casein (87.49%) [56,57].
4. The percentage of amino acids retained by the body after absorption through the intestines is known as Biological Value (BV). Values greater than 60% have been found for protein from *Lentinus lepidus*, *P. sajor-caju*, *P. ostreatus*, and *L. edodes*, harvested in Thailand [55].
5. The Protein-Digestibility-Corrected Amino Acid Score (PDCAAS) is a measure that provides information about the content and profile of amino acids compared to a reference protein, considering TPD as a correction factor [8]. Compared to meat and milk protein, with a TPD of 94% [58], edible mushroom protein typically has TPD values between 30% and 45% [55,57].

The cell wall of mushrooms contains a significant amount of non-digestible carbohydrates, composed of chitin and β-D-glucans as predominant components, along with mannan and other compounds [59]. These crude fibers cannot be decomposed in the human digestive tract due to the lack of specific hydrolysis enzymes that break glycosidic bonds. Even though they cannot be hydrolyzed, these types of fibers play a role in defecation and water absorption in the intestine [59]. In addition, β-glucans from edible mushrooms improve the immune system and have antitumor properties, such as ganoderan (*Ganodema lucidum*), grifolan (*Grifola fondosa*), lentinan (*Lentinus edodes*), pleuran (*Pleurotus ostreatus*), and schizophylan (*Schizophyllum commune*) [59]. The hydrolysis of non-digestible fibers in mushrooms can be improved through enzymatic digestion, [60] heat treatment, or other methods that also reduce certain allergic reactions caused by specific protein allergens in mushrooms [61]. In addition, the digestibility of proteins from macrofungi is also affected by compounds such as polyphenols and anti-nutritional factors [56], which inhibit intestinal absorption. This drawback could be avoided by obtaining protein isolates or hydrolysates from edible mushrooms [8].

The digestibility of proteins can be significantly improved by obtaining protein hydrolysates containing protein compounds with a lower mass, including amino acids, in addition to the initial unhydrolyzed protein. Protein hydrolysates contain polypeptides, oligopeptides, and amino acids, all of which are produced through controlled hydrolysis of internal peptide bonds or at the ends of protein molecules, depending on the hydrolysis process used (chemical with acids or bases or enzymatic with endo- or exopeptidases). Vioque et al. [62] showed that a partial hydrolysis of 5–10% increases protein solubility both due to a decrease in the molecular weights of the resulting compounds and to an increase in the number of polar groups that can form hydrophilic bonds with water molecules. Moreover, the initial configuration of the protein subjected to hydrolysis is drastically modified by the breaking of peptide bonds or physical interactions that stabilize it.

Vioque et al. [62] reported that protein hydrolysates with a degree of hydrolysis greater than 10% (considered by the authors to be extensive hydrolysates) can be used in the food industry for special products aimed at specific groups of people (athletes, growing children, people who need to increase or restore their muscle mass). In addition, protein hydrolysates appear to be hypoallergenic components, which could improve health when introduced into food products [63].

The main technique for analyzing the protein content of a product has remained the determination of total N using the Kjeldahl method or the Bradford protein assay [64,65,66].

#### 3.1.1. Applications in the Food Industry

One of the trends in valorizing mushroom proteins in the food industry is the addition of different mushroom parts, including those resulting from cutting, to fortify so-called “muscle food” products [67], in order to enhance their nutritional, therapeutic, and sensory value. Mushrooms can be added, for example, into cooked beef [68], pork sausages [69], chicken [70], tuna [71], or Turkish meatballs [72], both to improve nutritional value and physical–chemical and sensory properties, as well as stability [67]. Thus, dried *Pleurotus ostreatus* mushroom added to beef patties improved the protein content, water holding capacity, and certain physical properties (plasticity, juiciness) and sensory properties [73]. For beef burgers supplemented with *Agaricus bisporus*, changes in texture and moisture were observed [74]. Textural properties, viscoelastic behavior, heat resistance, and emulsion structure were enhanced by adding 2% *A. bisporus* powder to a beef meat emulsion [75], while meat-based dishes [76] had better nutritional value and a more intense aroma.

Mushroom powder, containing protein and other nutrients, is valorized in the food industry for the production of valuable products for human health, such as pasta, bread, biscuits, cakes, sauces, breakfast cereals, etc. [77,78].

Mushroom-derived proteins are increasingly studied due to their exceptional properties and have become increasingly present on the market for all kinds of food supplements, food additives in the form of extracts and powders, meat substitutes, and the preparation of vegetarian foods [12,67,79]. These proteins, considered innovative products, are subject to pre-market procedures based on scientific risk assessments. The Novel Food Regulation states that the main purpose is “a high level of protection of human health”, and therefore, mushroom proteins require authorization under the EU Nutrition and Health Claims Regulation (NHCR) or, similarly, approval by the U.S. FDA [80].

#### 3.1.2. Mushroom Protein Hydrolysates as Sports Nutrition or Therapeutic Foods

Protein hydrolysates are used to increase the protein value and functional qualities of foods, in the production of specialized foods, and to combat or mitigate malnutrition. Due to the fact that they are rapidly absorbed from the digestive tract compared to native proteins, they can be part of athletes’ diets [8]. It has been found that protein hydrolysates can increase muscle glycogen and muscle mass levels, with a significant anabolic effect on the muscles [81]. In addition, protein hydrolysates have a neuroprotective effect [82], antioxidant, inhibitory, and antiproliferative action [83], and hepatoprotective action [84] and can reduce lipid peroxidation [85]. Also, ACE (angiotensin-converting enzyme) inhibitory activity has been observed in the case of *Grifola frondosa* [86] and *Agaricus bisporus* [87] mycelium hydrolysates, resulting in a decrease in blood pressure. Although there is considerable variability in the effects of consuming mushroom protein hydrolysates, they represent a trend in the development of products specifically for sports nutrition or therapeutic foods.

#### 3.1.3. Safety and Allergenicity Assessment of Mushroom Proteins

Although mushrooms are considered some of the healthiest foods due to the presence of bioactive compounds and the quality of their proteins, some people may experience allergic reactions after consuming them. Currently, the mechanisms underlying the development of allergies are not fully understood, but some studies have shown that mushroom allergies may be due to a lack of serum copper oxidase in the body [88]. Other studies have explained these allergic reactions according to the presence of specific protein allergens in mushrooms, which can be reduced by various treatments, such as heat treatment, treatment with acids and bases, ultrasound, irradiation, and enzymatic digestion [61]. When assessing mushrooms proteins or foods containing these proteins, the issues raised by the uncertainty regarding acceptable risks and the evaluation criteria for determining the safety of the respective food must be taken into consideration. Food safety assessment guidance [89] considers allergenicity as a major hazard that may arise from the consumption of foods containing proteins. Proteins and foods containing these proteins can cause allergic reactions by inducing IgE production and an IgE-mediated immune response in the human body [90]. There is legislation in the EU [91] that describes the pre-market approval requirements for novel food and guidance on these requirements [89]. Food safety is defined in the Codex Alimentarius as “assurance that food will not cause harm to the consumer when it is prepared and/or eaten according to its intended use” (allergen/CAC/RCP), and in the US, the concept of “safe” and “safety” for food additives represents the “reasonable certainty in the minds of competent scientists that the substance is not harmful under the conditions of its intended use.” [90].

Currently, in Asian countries and the USA, the market commercializes a large number of food products and supplements containing mushrooms, while in Europe, these products are subject to strict regulations by the European Commission [91,92]. In fact, randomly harvesting wild mushrooms from different areas is dangerous in the first place because of relatively frequent mushroom intoxication due to insufficient knowledge of edible and poisonous species. Harvesting mushrooms from their natural environment is subject to restrictions, and their sale is prohibited, in accordance with guidelines or legislation for the safe commerce of wild mushrooms. All of these aspects differ depending on the area, country, mushroom consumption behavior, and culinary traditions [93].

In a mini-review, Fernandez et al. present the main methods for assessing the presence of allergens, namely in silico analysis (which uses the similarity between the primary amino acid sequence and an allergen database); in vitro analysis, which consists of protein stability measurements and immunological assays, e.g., ELISA and immunoblotting; and other methods, referring to in vivo studies using laboratory animals, particularly mice. These tests have their limitations due to both the type of analysis and the food matrix in which the allergen is present, the type of animal and diet, and other factors, which require further study and the need for validation of the methods [94].

### 3.2. Proteins with Functional Roles

**Figure 3:**
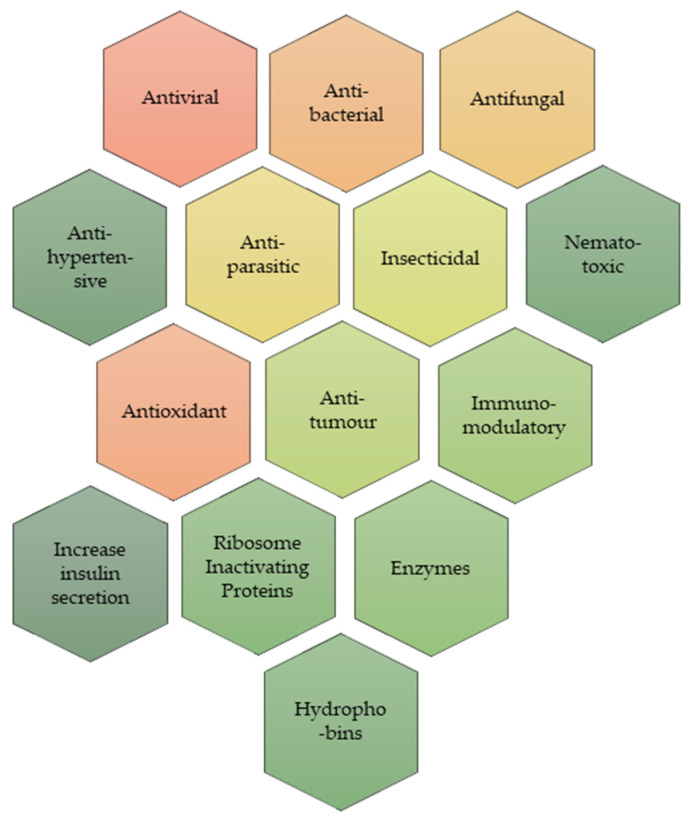
The main properties of mushroom proteins (own creation).

In addition to their structural role, proteins and peptides in macrofungi also have health benefits, such as antimicrobial, antiviral, antioxidant, anticancer, hypotensive, angiotensin-converting enzyme (ACE) inhibition, immunomodulatory, and enzymatic action (Figure 3).

This group of proteins includes the following categories: lectins (glycoproteins), immunomodulatory proteins, enzymes (laccase, cellulases, ribonucleases), ergothioneine [95], enzyme inhibitors, ribosome-inactivating proteins, and hydrophobins [96].

#### 3.2.1. Lectins

Lectins are proteins of nonimmune origin reversibly bound to specific sugars, precipitating polysaccharides, glycoproteins, and glycolipids to which they bind [36,97]. In other words, lectins play a role in biorecognition through their interaction with various compounds in the category of glycans and glycoproteins on the cell surface [98].

In 1910, the first fungal-origin lectin with toxic properties was discovered. Subsequent research showed that mushrooms synthesize significant levels of lectins, which, similar to plants, play a role in defense or in the process of mycelium differentiation into fruiting bodies. Lectins have been found in both mycelium and fruiting bodies [97,99]. Various studies have reported that extracts from the fruiting bodies of edible medicinal mushrooms such as *Agaricus pilatianus*, *Coprinus comatus*, *C. micaceus*, *Macrolepiota rachodes*, *Tricholoma fractum*, *Amanita ovoidea*, *Melanoleuca brevipes*, *Leucoagaricus leucothitus*, and *Lepista nuda*, as well as mycelial extracts from *Cerrena unicolor*, *Ganoderma ramnosissmum*, *Ganoderma lucidum*, and *Trametes versicolor*, contain lectin [100].

It has been shown that related mushroom species synthesize lectins with a similar structure and specificity, although the degree of variability is relatively high [36].

Lectins are composed of several identical or different subunits, between which weak noncovalent bonds are established. At present, numerous different lectins have been isolated and characterized in terms of their 3D structure, glycosylation, and carbohydrate specificity [36,96,101,102]. Lectins in mushrooms have molecular weights between 12 and 68 kDa [103] and bind to various carbohydrates, such as glucose, lactose, raffinose, turanose, N-acetyl glucosamine, and inulin [97].

##### Applications of Lectins

Numerous studies have shown that lectins in mushrooms have antitumor, mitogenic, antiproliferative, immunopotentiating, antidiabetic, hypotensive, and anti-HIV1 reverse transcriptase activities [104].

Antitumor role: Recently, numerous studies have demonstrated the role of mushroom lectins in the treatment of various forms of cancer based on their antiproliferative properties. Singh et al. [105] showed that lectins synthesized by species of *Pleurotus*, *Aleuria*, *Russula*, *Volvariella*, *Agrocybe*, and others acted on forms of cancer such as leukemia, carcinoma, sarcoma, and hepatoma. Also, mushroom lectins can work as biomarkers that, because of their remarkable specificity, can bind to tumor cells [106].

Yamasaki et al. [107] noted that lectins from mushrooms of the genera *Agaricus, Boletus*, and *Pholiola* have a tumor-inhibiting effect in colon cancer because they bind by cross-linking with sialyl-Lewis (a glycan on the surface of tumor cells). Other types of lectins synthesized by *Polyporus squamosus* and *Marasmius oreades* can inhibit protein synthesis in mammalian cancer tumor cells [107].

Lectins have been reported to cause pronounced inhibition of the proliferation of the human tumor cell lines HeLa, SW480, SGC-7901, MGC80-3, BGC-823, and HL-60 and mouse sarcoma S-180 S-180 tumor cells in vivo [108]. Lectins from *Pleurotus citrinopileatus* also inhibited sarcoma 180 in ICR mice, with an IC50 value = 0.93 μM [104].

Immunomodulatory role: Mushrooms synthesize four types of compounds with immunomodulatory action, including lectins, polysaccharides, terpenoids, and other proteins [109]. Due to their high specificity for certain sugars, lectins can reversibly bind glycosyl groups on the surface of lymphocytes, which results in a cascade increase in the cellular immune response. This leads to both their proliferation and an increase in their activity through phagocytosis and cytokine release. Lectins with these properties have been classified as fungal immunomodulatory proteins (FIPs), having considerable medicinal and therapeutic potential [36].

It has been reported that lectins from *Volvariella volacea* have more intense immunomodulatory action than that of concanavalin A [110]. Mushrooms from the species *Tricholoma mongolicum* synthesize two lectins that have been named TML-1 and TML-2, with immunomodulatory and antitumor action manifested in vivo [111], and have a positive effect on the synthesis of nitrite and tumor necrosis factor (TNF)-a but exert inhibitory action against mouse lymphoblast-like (p815) mastocytoma cells [111].

Sze, Ho, and Liu (2004) [110] explained the mechanism underlying the immunomodulatory action of lectins through calcium influx, the induction of CD25 and CD69 activation markers, cytokine production, and cell proliferation.

FIPs represent a new family of protein immunomodulators discovered in 1989 in the species *Ganoderma lucidum* and named Ling-Zhi-8 [112]. Although not all FIPs are similar to lectins, they have some properties similar to them. Numerous species synthesize FIPs: *Flammulina velutipes*, *Ganoderma tsugae*, *Ganoderma sinensis*, *Poria cocos*, *Volvariella volvacea*, *Antrodia camphorate*, *Ganoderma japonicum*, *Ganoderma microsporum*, and *Trametes versicolor* [113,114,115,116,117,118,119,120]. All of these FIPs have very similar amino acid sequences and dimeric structures. According to studies, FIP-fve produced by *Flammulina velutipes* has a positive effect on mitogenesis in human peripheral lymphocytes [113]; FIP-gts produced by *Ganoderma tsugae* induces cytokine secretion and has a positive effect on IFN-g expression [114]; and FIP-glu from *Ganoderma lucidum* enhances the transcription of interleukin IL-2, IL-3, and IL-4, interferon IFN-g, and tumor necrosis factor TNF-a [121]. FIPs are also used in tumor immunotherapy and suppress tumor invasion and metastasis [122,123].

Lectin from *Agaricus bisporus* is able to activate TNF-α and nitric-oxide-producing RAW 264.7 macrophages [124]. Li et al. [120] reported that lectins from *Trametes versicolor* induced an increase in the proliferation of lymphocytes in human peripheral blood, which led to an increase in macrophage-induced alpha tumor cell necrosis in mice.

Through this pathway, antitumor effects, as well as the inhibition of diabetes in autoimmune processes, are also mediated [105,125,126].

Mitogenic potential: Another characteristic of mushroom lectins is their mitogenic potential, as they are capable of transforming small resting cells (lymphocytes and splenocytes) into large blast-like cells ready for mitosis [127].

Antioxidant activity: Some studies have demonstrated that mushroom lectins have significant antioxidant activity, which could be used to inhibit forms of oxidative stress in cells, which could also represent ways to treat cancers [128].

Antiviral activity: Due to their characteristics, lectins can bind to various glycoproteins located on the surface of viruses, which can no longer bind to cellular receptors for the virus. Lectins can also bind to the active site of viral polymerases [129], inhibiting viral replication in the host cell. The antiviral activity of lectins has been proven against herpes simplex types 1 and 2, hepatitis C, influenza A/B, Japanese encephalitis virus, HIV, SARS virus, and the current SARS-CoV-2 virus [128].

Antinematode action: In addition, some lectins have nematotoxic action [34,130] and are used in the composition of “glycan-based” vaccines, such as the vaccine against the *Haemonchus contortus* parasite that infects some ruminants.

Numerous studies have shown that lectins from macrofungi contribute to lowering blood glucose concentrations. In the species cordyceps (*Ophiocordyceps sinensis* (Berk.)), a peptide has been discovered that lowers blood glucose in alloxan-induced hyperglycemic rats when administered at a dose of 50–100 mg/kg of body weight [131].

Other uses: Due to their properties and high specificity, lectins are also used in various clinical diagnostic analyses [96], such as the analysis of altered glycosyl groups in tumor modifications, in some neurodegenerative diseases, and in some microbial infections. Some affinity chromatography techniques involve establishing bonds with glucoconjugates from different sources (plasma glycoproteins, glycans on the surface of bacteria or on the surface of stem cells, or recombinant therapeutic glycoproteins) [96].

Various techniques such as lectin microarrays and lectin-based biosensors are based on the potential of lectins to bind carbohydrate groups in a specific and selective manner.

#### 3.2.2. Enzymes

Numerous enzymes, especially from the hydrolase and oxidoreductase classes, are synthesized by fungi during primary and secondary metabolism to release nutrients from external substrates. These enzymes are used industrially to treat natural polymeric substrates such as lignocellulosic materials, starch, proteins, and others.

Basidiomycota are mainly known for the biosynthesis of laccases, enzymes that break down lignocellulosic material from different substrates, acting on phenolic compounds, aromatic amines, azo dyes, aromatic hydrocarbons, and other compounds. Laccases are synthesized in mushroom cells for cell protection, sporogenesis, and pigmentation, as well as to supply nutrients from the environment. Laccases are synthesized together with lignin peroxidase and manganese peroxidase and are used industrially for the treatment of various lignocellulosic wastes and by-products and in the food industry for the extraction and clarification of fruit juice, wine stabilization, in baking, and in the production of sugar beet pectin. In the textile industry, laccases are used for fiber biobleaching and denim washing, and in the paper and pulp industry, laccases are active in the processes of pulp delignification and deinking of paper. Recently, these enzymes have been valorized in polymer synthesis and green nanoparticle synthesis, as well as in the construction of biosensors. The decomposition of lignocellulosic substrates used in biogas production or in various fermentation processes is improved by the addition of laccases [132]. *Pleurotus ostreatus*, *Lentinula edodes*, *Ganoderma sp.*, *Phlebia radiata*, *Trametes versicolor,* and others are some of the most effective basidiomycete species in the process of lignocellulosic material degradation [133,134,135].

Other enzymes produced by Basidiomycota are those in the hydrolase class, which include amylases, proteases, cellulases, hemicellulases, pectinases, xylanases, and others, which can act together, allowing for more advanced degradation of complex substrate from plant materials [37,136].

Another enzyme in the oxidoreductases class is tyrosinase, which is involved in the synthesis of melanin pigment, which causes post-harvest browning, with this enzyme being described by [137] in *Agaricus bisporus*. Tyrosinase from mushrooms catalyzes the reaction of forming diphenols from monophenols and is used for the production of antioxidants as additives in the food and pharmaceutical industries [138,139]. In the medical field, due to its action and similarity to mammalian tyrosinase, tyrosinase is used in the study of melanogenesis [95] but also for the treatment of tumors and Parkinson’s disease [140]. *Agaricus bisporus* synthesizes a tyrosinase that has been studied for the construction of a biosensor for dopamine [12] and phenolic pollutants in the environment [13].

Another enzyme of interest is phytase, used in the food industry as an additive for the degradation of phytates and the improvement of phosphorus and mineral uptake [141].

Basidiomycota can synthesize proteases used in industry for the hydrolysis of various proteins and milk coagulation (aspartic proteases for milk-clotting properties), meat tenderization, protein hydrolysates, and medicine (metalloproteases with fibrinolytic activity) [142].

In addition, mushrooms synthesize a series of protease inhibitors, which have been characterized and grouped into several general types. Two families of cysteine protease inhibitors [143,144] with properties specific to Basidiomycota have been described, different from similar compounds in plants and animals. Two types of serine protease inhibitors have also been reported, one represented by subtilisin-like proteases from *Pleurotus ostreatus* and a trypsin-specific inhibitor from *Coprinopsis cinerea* [145,146]. The unique properties of the highly specific protease inhibitors synthesized by Basidiomycota are especially valorized in the medical field.

Another category of hydrolytic enzymes with beneficial activities in the medical field is ribonucleases, which catalyze RNA hydrolysis, an activity that gives them recognized antitumor and antiviral properties [147].

#### 3.2.3. Ribosome-Inactivating Proteins (RIPs)

Ribosome-inactivating proteins (RIPs) are a category of enzymes that act on the rRNA molecule by catalyzing the hydrolysis reaction of one or more adenosine residues, which inhibits protein synthesis at the ribosome level. This activity in RIPs can be valorized to combat cancer. Such RIPs are considered marmorin and hypsin from *Hypsizygus marmorus*, calcaelin from *Calvatia caelata*, and lyophyllin from the fruiting body of *Lyophyllum shimeji* [148]. Some of these RIPs (marmorin and hypsin) have shown cytotoxic activity against hepatocellular carcinoma, breast cancer cell lines, and human leukemia [149].

#### 3.2.4. Hydrophobins (HPs)

Hydrophobins (HPs) are surface-active, amphipathic proteins synthesized only by fungi, with a role in decreasing surface tension for hyphae growth, attachment to hydrophobic substrates, insect invasion, and endophytic association with plants [150]. They are small proteins, consisting of 100–150 amino acids, specifically containing 8 cysteine molecules. Due to their dual hydrophobic and hydrophilic properties, these molecules can act at liquid–liquid or liquid–air interfaces, such as water–air, air–oil, and water–oil interfaces [151]. The amphipathic nature of hydrophobins is valorized in various industrial applications where they function as surfactants: surface coatings, the formation of dispersed mixtures of hydrophobic materials–water, foam stabilization, biosensors, and others.

HPs were first isolated from *Schizophyllum commune* and later were found in *Agaricus bisporus* [152,153,154], *Pleurotus ostreatus* [155,156], *Pleurotus nebrodensis* [157], *Dictoynema glabratum*, [158], and *Tircholoma terreum* [159], for which multiple genes encoding these proteins have been discovered. Most of these genera are GRAS (Generally Recognized as Safe) [151].

Although industry interest in these proteins is high, laboratory production has shown that wild strains rarely synthesize hydrophobins in submerged cultures [160], which would recommend the use of genetically modified strains and further research. Wösten et al. [161] showed that *Agaricus bisporus* produces three hydrophobins, namely ABH1, ABH2, and ABH3, but in a laboratory culture medium, only ABH3 is synthesized, where a yield of 2 mg/L was obtained [153].

Coating biosensors with a hydrophobic coating helps to avoid denaturation of the immobilized proteins used and contributes to the stabilization of suspensions. Martínez et al. [156] reported that the use of a hydrophobic protein from *Grifola frondosa* had a positive effect on the solubility of carbon nanotubes and on the immobilization of antibodies.

## 4. Cultivation of Mushrooms from the Basidiomycota Phylum

The traditional cultivation of edible mushrooms is widespread throughout the world, but lately, mushrooms are increasingly being grown as mycelium in submerged culture (SC) or solid-state cultivation (SSC) systems [162], depending on the species used, the compound of interest, conditions, equipment, and costs. Traditional mushroom cultivation is subject to the influence of various environmental factors, which causes large fluctuations in the quality of the final product, the homogeneity of the batches, and the type and percentage of compounds of interest. Traditional cultivation of fruiting bodies can extend over several months, and the quality of the final product is not always reproducible [163,164,165].

In addition, this type of crop can be damaged by pests, microorganisms, viruses, insects, or other organisms, which cause considerable damage.

Cultivating mushrooms using different culture substrates eliminates some of these disadvantages and can be integrated into the circular economy by using substrates representing by-products from the agri-food industry or other sectors. The main advantages of SCs and SSC systems are shorter mycelium growth times; more rigorous control over the growing conditions; a considerable decrease in the presence of unwanted organisms; more diversified possibilities for processing the obtained biomass, including the extraction of certain components; and, last but not least, the possibility of using new hyperproductive species resulting from genetic modifications. Reducing the risk of contamination leads to obtaining products that are safe for consumption, both for the human diet and for the medical field [166,167]. All of these could have a significant impact on the final product and the environment.

Submerged cultivation uses liquid media containing various nutrients, into which oxygen is dispersed through agitation [168]. Recent studies have shown that the biosynthesis of useful metabolites from fungi is similar in mycelium to that in fruiting bodies [15], and Basidiomycota can be successfully cultivated in bioreactors. The most commonly used bioreactors in mushroom cultivation are stirred tank bioreactors and air-lift bioreactors (which preserve the structure of mycelium pellets), with macrofungi development taking place in batch cultivation, but also fed-batch cultivation and repeated fed-batch cultivation [166,169,170,171].

**Figure 4:**
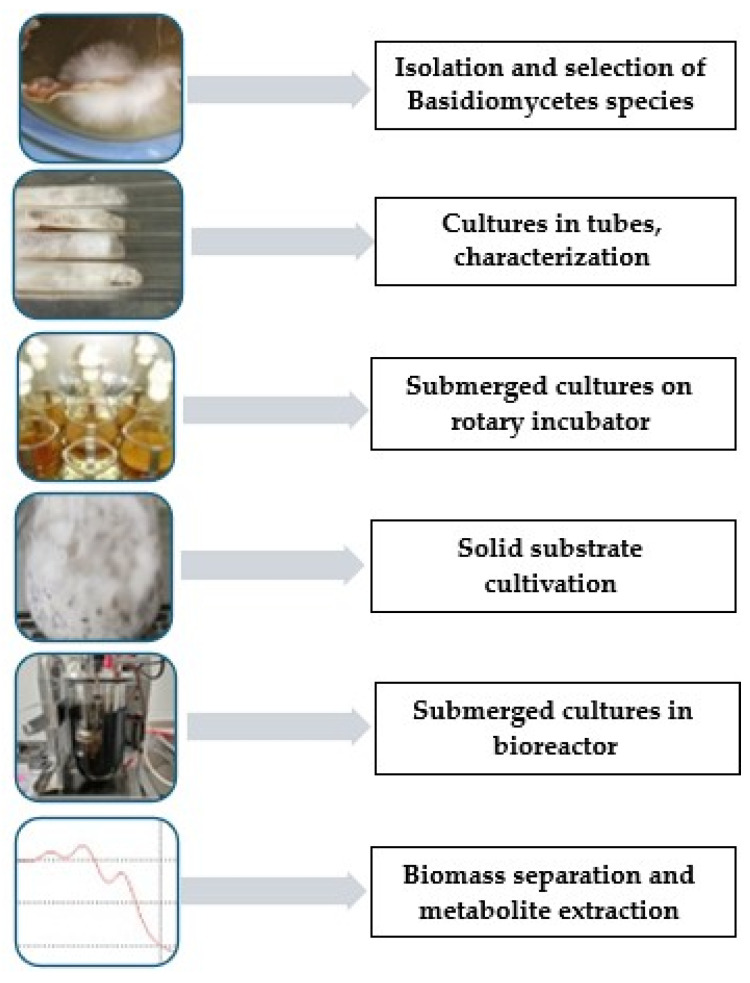
Stages of mycelial biomass production from mushrooms (own creation).

For the cultivation of mycelium in the laboratory or at the industrial level in bioreactors, it is necessary to follow several stages that allow for the optimization of the process, the characteristics of the final product, and the costs. In addition, for the pharmaceutical and medical fields, products with a minimal microbial load can be obtained. The stages of the mycelium culture process are as follows (Figure 4):

1. The isolation and selection of mushroom species with high production potential in terms of the compound of interest;
2. Obtaining and maintaining the laboratory stock culture and choosing a method to preserve the properties of the species;
3. Testing the cultivation conditions at the laboratory level (it is necessary to choose the optimal culture medium, temperature, pH, type and quantity of inoculum, aeration and agitation, culture duration, etc.);
4. Cultivating the fungus in bioreactors of different capacities (the type of bioreactor and cultivation parameters will be chosen);
5. Separation of the product with a high protein content is usually achieved by processing the mycelium and through extraction, centrifugation, precipitation, and other methods [172], along with analyzing the synthesized compounds.

Most studies on the cultivation of edible mushrooms using SCs have focused on the genera *Pleurotus*, *Agaricus*, *Lentinus*, *Cordyceps*, *Morchella*, and *Tuber* [173]. Among these, particular attention has been given to the species *Pleurotus ostreatus* [174], *Pleurotus eryngii* [175], and *Pleurotus pulmonarius* [176,177].

An SC of *Lentinus* was studied by Assis et al., 2013 [178], for the synthesis of antitumor compounds. In addition, the effect of the culture medium containing agri-food by-products and plant growth hormone supplements (indole-3-acetic acid, gibberellic acid, and kinetin) on the amount of mycelium produced was demonstrated [179,180]. The best results were recorded for indole-3-acetic acid, which resulted in a more than 4% increase in the protein content [181].

*Pleurotus sajor-caju* was cultivated in a liquid medium with glucose, and it was demonstrated that the amount of protein in the mycelium can exceed the amount of protein in the fruiting bodies [179]. The highest amount of protein, between 40 and 49%, was observed for this species when corn stover was used as the substrate [181].

With regard to enzyme biosynthesis, numerous studies have shown that some fungi produce higher amounts of enzymes in submerged culture. For example, *Pleurotus dryinus* in a liquid medium containing tree leaves produces cellulase with five times higher activity than that in solid-state cultivation [182]. Bentil et al. [168] report that white-rot basidiomycetous fungi synthesize higher amounts of cellulolytic enzymes in liquid media containing carboxymethyl cellulose as a carbon source than in solid media culture. Laccase was obtained through submerged cultivation of Basidiomycete species from the genera *Pleurotus* and *Agaricus* [183,184].

Usually, the production of edible mushrooms is based on the domestication process of valuable strains that have been selected from the natural environment. Although not all natural mushrooms can be successfully cultivated and some strains diminish in their productivity over time and change in their production or therapeutic properties, the results obtained can be significant. For this purpose, according to research, several steps must be taken, namely strain isolation, establishing the optimal conditions for mycelia growth, testing substrates and culture media for SSC or SC, optimizing the culture parameters, obtaining and processing the product, and characterizing its nutritional and other properties. The most commonly domesticated strains for cultivation belong to the temperate zone, such as Agrocybe [185] and Macrocybe [186] species, although lately, numerous studies have focused on tropical species [187], such as *Pleurotus giganteus* [187], *Ganoderma lucidum*, *Hericium erinaceus* [187,188], and *Agaricus subrufescens* [189]. The discovery and domestication of wild strains are not simple and usually require patience, lengthy studies, and skill and innovation.

## 5. Future Trends

**Figure 5:**
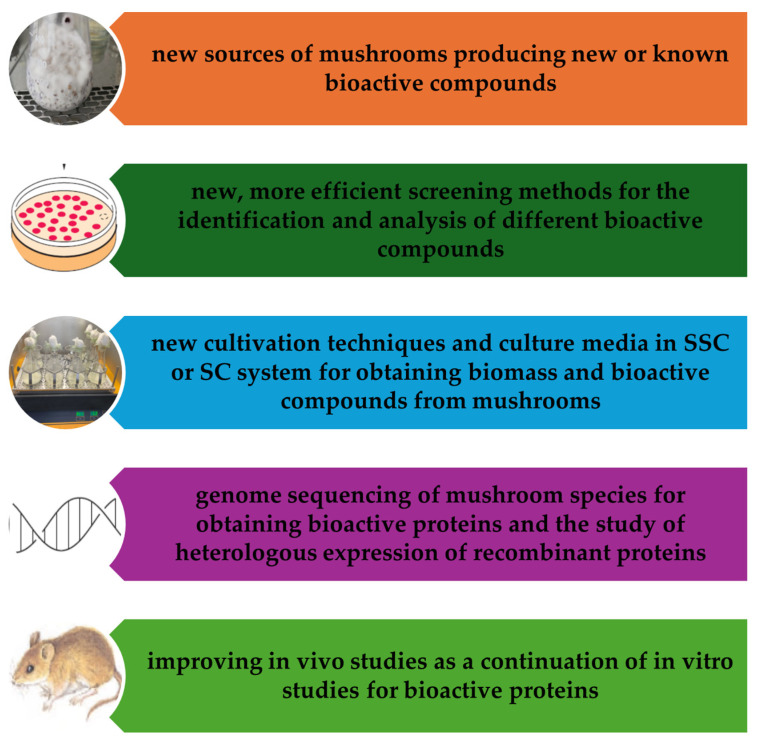
Future trends and perspectives (own creation).

The main trends in protein biosynthesis from Basidiomycota relate both to improving the properties of the producing strains and to optimizing the cultivation methods in submerged culture and on solid substrates (Figure 5).

The use of new strains with a high production potential is achieved through selection and the use of new screening tests to identify new types of proteins with applications in medicine, industry, and the environment. Future studies should focus on cultivating selected wild mushrooms characterized by nutritional value, therapeutic properties [190], high productivity, and stability.

The process of domesticating mushrooms is being intensively studied with the aim of increasing productivity and the quality and quantity of bioactive metabolites in fruiting bodies and mycelium [191,192,193,194].

The development of proteomics can provide various advanced methods for the analysis of cellular proteins, as well as new separation techniques. There is a tendency to valorize various inexpensive agri-food by-products, especially those of a lignocellulosic nature, as components of culture media for a sustainable biosynthesis process.

The techniques for the analysis, extraction, separation, and characterization of various proteins and bioactive compounds developed by genomics, proteomics, and metabolomics promise remarkable results in research on edible mushrooms [195]. Other techniques for improving the growth of edible mushrooms involve exposure to ultraviolet radiation [196], gamma rays [197], and N^+^ ion beams [198] and obtaining mutant and hybrid strains that can be crossbred further with other selected strains [173,193].

Sequencing the genome of interest for fungi represents an efficient tool for investigating new types of proteins, characterizing them, producing them, and testing them in various applications. This technique is related to the production of hyperproductive strains and the biosynthesis of recombinant proteins. One of the most studied techniques is CRISPR/Cas9, which is much more efficient and accurate than the outdated methods for inducing mutagenesis. This technique has been used, for example, to regulate multiple genes associated with protein secretion in *Ganoderma lucidum* [199] and as a modern breeding technique in *Lentinula edodes* [200] and *Pleurotus eryngii* [201]. Another approach in the study of improving the properties of edible mushrooms remains the development of artificial breeding techniques [202] to obtain strains that can be cultivated as mycelium for industrial-scale use, using laser or molecular breeding techniques, including multiplex gene editing and targeting tools [199,202,203].

Although there are numerous bioassay guidelines for testing the bioactive compounds synthesized by fungi, further development of these techniques is needed in the future [165,204,205,206]. In order to obtain convincing and comparable results, these techniques should be fully standardized [207,208,209,210,211]. These techniques for analyzing bioactive compounds (through advanced approaches such as genomics, proteomics, and transcriptomics) must be constantly updated [22] so that they can also determine the health risk associated with the studied mushroom given that the nutritional and medicinal potential of most mushrooms has not been fully studied, especially in terms of its mechanisms of action at the cellular level [79,212].

Another trend concerns the isolation of highly stable proteins that are resistant to various environmental factors and have industrial applications.

Obtaining immobilized enzymes with superior characteristics, greater stability and lower cost, along with the valorization of mushroom hydrophobins for this purpose, represents a promising direction of research [81].

In vitro studies of mushroom protein properties should be continued with research into their action in vivo for clear and representative results. Although numerous studies have been conducted on laboratory animals, the properties of proteins related to the mode of administration or the food matrix in which they are introduced require further study [9].

Although numerous studies have been conducted on the cultivation of mushrooms in the form of mycelium in submerged cultures or on solid substrates, there are still many things that remain unclear or need to be optimized, such as the cultivation parameters, the construction of bioreactors for cell-sensitive cultures, and biomass processing [81].

Protein isolates and hydrolysates can be used in a wide range of applications, but several important knowledge gaps still need to be addressed. Among the issues that need to be addressed are the bioavailability of these protein isolates and hydrolysates, as well as aspects related to their safety in use (potential allergies, long-term use, and the presence of contaminants). Although protein isolates and hydrolysates obtained from edible mushrooms are generally considered safe, they are considered “novel foods” in the EU and require EFSA authorization before being marketed [213]. In the USA, these products can be classified as food supplements, but the producers have the responsibility of ensuring safety and conformity with the FDA guidelines regarding the labeling of such products [91]. In order to ensure the safety and efficacy of these proteins in the future, it is necessary that interdisciplinary research be conducted and that uniform regulatory approaches be adopted.

The limitations in obtaining bioactive compounds cover several aspects, namely cultivation and increasing productivity, the quality of these bioactive compounds, processing the biomass obtained in order to preserve its initial properties as much as possible, and analyzing toxic compounds or allergens. Last but not least, the degree of acceptability of mycelial biomass, extracts, and protein hydrolysates from mushrooms should also be taken into account. In the case of obtaining new mushroom strains or isolates, extracts, and protein hydrolysates, these are considered novel foods and are subject to the regulations in force [89,214,215,216].

## 6. Conclusions

Due to their impressive nutritional profile and high adaptability, edible mushrooms represent great promise in the future of food and medicine, possessing a wide variety of versatile proteins that satisfy the requirements of eco-friendly nutrition [195]. Furthermore, mushroom cultivation, both traditional and submerged, has implications in sustainable waste management due to the valorization of waste and by-products, especially those of agri-food origin, in the transformation of unused organic matter into high-value biomass rich in protein. Last but not least, the valorization of enzymes from the group of laccases, cellulases, hemicellulases, proteases, and others brings significant benefits to the agri-food industry and the environment. Through all these properties, the valorization of mushrooms fits into the development of an expanding circular economy.

Edible mushrooms represent both a promising nutritional and functional source of protein and, at the same time, a relatively unexplored one, even though they have been known and consumed for so long. Cultivated either as fruiting bodies or in the form of mycelium, edible mushrooms synthesize a multitude of compounds with nutritional and functional value, namely polysaccharides, proteins, lipids, phenolic compounds, terpenes and terpenoids, nucleotides, vitamins, and minerals.

The group of proteins includes the following categories: lectins (glycoproteins), immunomodulatory proteins, enzymes (laccase, cellulases, ribonucleases), enzyme inhibitors, ribosome-inactivating proteins, and hydrophobins.

Proteins and peptides in mushrooms have both a nutritional and functional role, with numerous health benefits, such as antimicrobial, antiviral, antioxidant, anticancer, hypotensive, angiotensin-converting enzyme (ACE) inhibition, immunomodulatory, and enzymatic activities.

Mushroom cultivation as mycelium can be realized both on solid substrates and in submerged culture, followed by protein separation and extraction. The production of edible mushrooms in artificial conditions has a number of advantages over traditional cultivation, but it depends greatly on the species used, the environment, and the cultivation parameters. Expanding and increasing the production of edible mushrooms under artificial conditions would be one way to alleviate some of the world’s malnutrition and medical problems, contributing to improved food security.

Due to their properties, relatively simple cultivation methods, and abundance of proteins and bioactive compounds, edible mushrooms represent an important source of nutrients in the future of food and can be considered the need of the hour in the food and pharmaceutical industries.

## Data Availability

No new data were created or analyzed in this study. Data sharing is not applicable to this article.
